# The Zooarchaeology of ancient whaling practices in Portugal: A review and a new Roman Republican contribution at Castelo Velho de Safara

**DOI:** 10.1371/journal.pone.0310215

**Published:** 2024-11-20

**Authors:** Mariana Nabais, Rui Soares, Youri van den Hurk

**Affiliations:** 1 IPHES-CERCA—Institut Català de Paleoecologia Humana i Evolució Social, Zona Educacional 4, Campus Sescelades URV (Edifici W3), Tarragona, Spain; 2 Departament d’Història i Història de l’Art, Universitat Rovira i Virgili, Tarragona, Spain; 3 UNIARQ—Centro de Arqueologia da Universidade de Lisboa, Faculdade de Letras, Universidade de Lisboa, Lisbon, Portugal; 4 Câmara Municipal de Silves, Silves, Portugal; 5 Department of Archaeology and Cultural History, NTNU Vitenskapsmuseet, Norwegian University of Science and Technology, Trondheim, Norway; 6 UMR 7209 Archéozoologie, Archéobotanique—Sociétés, Pratiques et Environnements (AASPE), Centre National de la Recherche Scientifique (CNRS), Muséum National d’Histoire Naturelle (MNHN), Paris, France; Liverpool John Moores University, UNITED KINGDOM OF GREAT BRITAIN AND NORTHERN IRELAND

## Abstract

The identification of archaeological cetacean specimens to the species level often presents challenges, primarily due to the frequent fragmentation of their remains. This limitation hampers our ability to comprehensively understand the spatiotemporal distributions of cetaceans in the past and constrains our knowledge of early whaling activities. To address this issue, a systematic zooarchaeological review was conducted, encompassing published literature and reports that compile available data related to cetaceans retrieved from archaeological contexts in Portugal, spanning from the Middle Palaeolithic to the 18^th^ century. Furthermore, this study introduces a new discovery of a cetacean specimen from the Roman Republican levels at Castelo Velho de Safara, which has been incorporated into the aforementioned dataset. The taxonomic identification of this specimen was accomplished using Zooarchaeology by Mass-Spectrometry (ZooMS). The study confirms that cetacean remains have been present in Portuguese waters since the Middle Palaeolithic, with higher concentrations in the southern and central regions of the country. ZooMS analysis confirmed the presence of the North Atlantic right whale at the inland site of Castelo Velho de Safara, which supports whale product trading during the Roman Republican period in Portugal. Evidence of cetacean exploitation continues to the Medieval and Modern periods, reflecting a strong cultural link between Portuguese ancient culture and whaling practices.

## 1. Introduction

Assessing the role of archaeological cetaceans–i.e. remains of whales, dolphins and porpoises found in archaeological contexts–is a complex process and it involves a multi-disciplinary approach that combines biological, historical, and ethnographic data to investigate the cultural significance of these marine mammals within past societies. Cetacean biology and ethology can shed light on the ecological and environmental factors that may have influenced human-cetacean interactions. For instance, the presence of certain cetacean species may have induced the development of local whaling practices, or the creation of myths and legends. These can be further explored through the analysis of historical records and cultural artefacts, in order to better understand how these animals were perceived and used by humans in different time periods and different cultural contexts [1, 2]. Such views can be complemented by the use of ethnography, which can provide insights into the cultural significance of cetaceans in more recent societies. In addition to these methods, archaeological cetacean remains can also offer valuable information through detailed zooarchaeological and taphonomic analyses, and through new biomolecular techniques, such as Zooarchaeology by Mass Spectrometry (ZooMS). ZooMS has proven crucial for identifying species in fragmented archaeological cetacean remains, which are often indistinguishable osteologically and lack comprehensive reference collections [3]. However, a preliminary step to address these challenges involves analysing the current distribution and abundance of cetacean species in a particular territory. Currently, the waters of Portugal are home to a diverse range of cetacean species. Based on data provided by Augusto et al (2012) [4], Castro et al (2022, 2024) [5, 6], Correia et al (2019) [7], Santos et al (2007) [8], among others, the waters around the Portuguese mainland host a resident population of bottlenose dolphins (*Tursiops truncatus*) in the River Sado estuary and frequent visitors in the Algarve region, where common dolphins (*Delphinus delphis*) are also often observed. Sightings of the striped dolphin (*Stenella coeruleoalba*), the Risso’s dolphin (*Grampus griseus*), the fin whale (*Balaenoptera physalus*) and the common minke whale (*Balaenoptera acutorostrata*) are common in that region. Harbour porpoises (*Phocoena phocoena*), killer whales (*Orcinus orca*), pilot whales (*Globicephala melas*) and, less frequently, humpback whales (*Megaptera novaeangliae)* are also observed. North Atlantic right whales (*Eubalaena glacialis*) were once commonly found near the coast until overexploitation drastically reduced their numbers by the late 17th century, leading to their eventual extirpation from the eastern North Atlantic by the mid-20th century [9, 10].

However, contemporary distributions may not necessarily align with the historical habitats and migration routes. This is due to thousands of years of whaling activities in European waters that changed the composition and the range of whale populations, as well as their ecosystems, especially the severe impact of industrial whaling of the 20^th^ century [9, 11–14]. Despite recent research developments in past cetacean populations [e.g. 15, 16], archaeological remains are still of key importance to better assess their original ecology and their interaction with human societies, but there are not many cetacean studies from archaeological sites. Additionally, most cetacean bones seem to be highly incomplete, damaged or altered due to being heavily processed, used as raw materials, or due to taphonomic processes. It can also be difficult to identify and recover cetacean remains from archaeological contexts in areas with very acidic soils, or with high levels of water saturation. Cetacean bones are also often poorly preserved due to their high fat content, which can lead to rapid decomposition and loss of bone integrity, compounded by the porous composition of bone tissue, especially the vertebra [15]. Consequently, most skeletal parts tend to lack important diagnostic features further challenging the identification process [17]. Finally, and based on past evidence and ethnographic accounts, most cetaceans from pre-industrial chronologies must have been processed on the beaches due to the large size of the animals [18, 19], as transporting a large cetacean to another location would have been as challenging as it is today (see for example [20]). As a result, in pre-industrial societies, only a small part of the carcasses would have been transported to archaeological sites, while the remaining skeletal parts would have been left out on the beach and washed away by the tides, making it almost invisible evidence to archaeologists [18, 19].

This study aims are threefold. Firstly, the remains of archaeological cetaceans from Portugal are reviewed in order to identify and assess their use and exploitation in different chronologies. Secondly, a new cetacean contribution recovered from the Roman Republican levels of Castelo Velho de Safara and identified through Zooarchaeology by Mass Spectrometry (ZooMS), is added to the list of archaeological cetacean remains. Thirdly, the origin of the Portuguese cetacean remains is discussed–whether they were obtained from active whaling, or by opportunistic scavenging stranded animals–, and if they were mostly used as a food resource, or as byproducts of other activities. The combination of zooarchaeological, biological, and historical data will give insight into which species were available and exploited along the Portuguese Atlantic coast, and can maybe hint at socio-economic stratification of those who engaged in cetacean use and exploitation in the later periods. Additionally, the distance from the sea will be considered to further understand the spatial dynamics of these activities.

### 1.1. Castelo Velho de Safara

**Fig 1:**
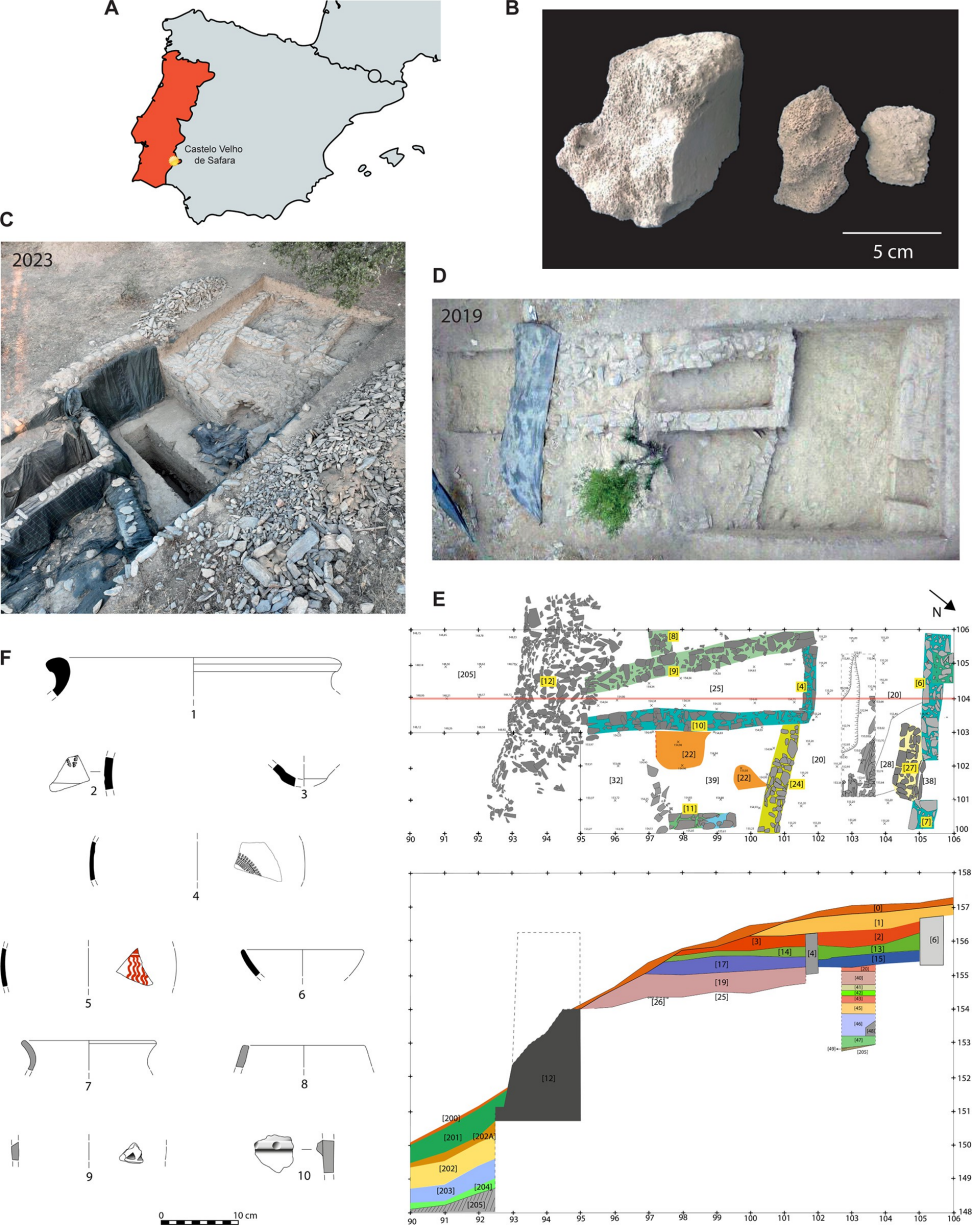
Archaeological discoveries at Castelo Velho de Safara. A) The site’s location map. B) Examples of some of the very fragmented cetacean remains recovered from Compartment 1. C) Aerial view of Trench 1 and its northern expansion in July 2023, featuring the rectangular test pit in the middle of the Roman Republican street area (photo by João Vidigal). D) Aerial view of Trenches 1 and 2 in July 2019 (photo by João Vidigal). E) Trenches 1 and 2 layouts with a red line marking the stratigraphic profile represented underneath. F) Examples of diagnostic pottery fragments recovered from stratigraphic level 19 in Compartment 1, including a large storage container (1), stamped pottery (2), a base of local/regional amphora (3), grey ware decorated with roulette (4), painted pottery (5), bowl of common ware (6), handmade pottery pans (7, 8), handmade pottery decorated with excised triangles (9), handmade pottery with impressed cord design (10).

The site of Castelo Velho de Safara (Moura, southern Portugal; 38°08’25”, 7°13’08”; Fig 1) is located on a rocky outcrop at the confluence of the Safareja stream with the Ardila river. The settlement presents a Chalcolithic occupation phase, followed by an extended hiatus of occupation since only by the middle of 1^st^ millennium BCE a new settlement was built. The latter corresponds to an occupation of the Late Iron Age, which extended over to the Roman Republican period and to the early days of the Empire (1^st^ century BCE). Castelo Velho de Safara has been systematically excavated since 2018, when Trench 1 was opened perpendicular to the thick defensive wall that was visible on the east part of the settlement, in the escarpment that faces the Safareja stream. The continuation of the archaeological works exposed the presence of a street running parallel to the defensive wall, showing a residential area on its north-western side, and two compartments on its south-eastern side, Compartment 1 and Compartment 2 [21–23]. The former is the one we will be focusing on as part of this study, and it may correspond to a storage facility attached to the inner part of the defensive wall. The material culture recovered in Castelo Velho de Safara has been confirming the four different occupation phases of the site, even though the best represented chronologies are those of Roman Republican age, as well as the beginnings of the Roman Empire, with a particular focus on materials dating from the mid- and late 1^st^ century BCE. This is mostly due to the presence of Italian red-gloss ware known as *terra sigillata*, Italian black-gloss ware and their local imitations on grey common ware, thin-walled vessels, Kuass ware, stamped and roulette decorations, as well as several rims and handles of Roman Republican ovoid and Haltern 70 amphora [21–23].

Focusing on Compartment 1 and its stratigraphy (Fig 1), the oldest deposition is the stratigraphic level 19, where the cetacean remains were recovered, followed by levels 17, 14 and the most recent deposition that is level 3. The diagnostic pottery materials recovered are presented in Table 1 and they reflect two different phases. The most recent phase, and easier to characterise, is found in levels 3 and 14 since it is defined by a package of imported materials, such as amphora from the Guadalquivir (in Spain), Italian black-gloss ware and its Iberian imitations, thin-walled vessels, clay lamps and Italian *terra sigillata*. These are materials typical of the 1^st^ century BCE, of late Republican age, indicating the presence of a Roman army on site, which is also supported by the recovery of several slingshot bullets from surface levels during a systematic survey of the site carried out in 2019. The Italian *terra sigillata* from level 3 dates from the early days of the Roman Empire, when the site was abandoned. Level 17 can maybe correspond to an intermediate phase, between the most recent (levels 3 and 14) and the later one (level 19). This is because it presents a scarce presence of painted pottery similar to levels 3 and 14, but a high quantity of handmade ware similar to level 19. The oldest phase of Compartment 1 is reflected in the materials recovered from level 19, which is associated with the foundation levels of the Roman Republican building and the street that runs parallel to the settlement’s defensive wall. All imported pottery is absent from this level showing, instead, a large presence of painted pottery, handmade vessels and two fragments with roulette decorations, which points towards an early 1^st^ century BCE chronology.

**Table 1:** Diagnostic pottery materials recovered from Compartment 1 of Castelo Velho de Safara. The finds are categorised by stratigraphic levels, providing a preliminary inventory with the Minimum Number of Individuals (MNI) indicated at the bottom of the table. Frag = fragments.

Diagnostic pottery types	Stratigraphic Levels			
	19	17	14	3
Wheel-made bowl	23	12	14	27
Grey bowl	2	2	3	-
Painted dish/bowl	2	2	-	3
Handmade bowl	2	-	-	.
Wheel-made pot	30	25	23	50
Painted pot	42	14	13	18
Handmade pot	11	8	4	4
Pot with plastic decoration	1	1	-	1
Cord with imprint	1	1	-	1
Stamped decoration	1	1	2	-
Roulette decoration	2	-	2	3
Fenestrated ware	-	-	1	-
Amphora	-	2	6	6
Lid	-	-	3	2
Italian black-gloss ware	-	-	1	3
Imitation of Italian black-gloss ware	-	2	8	6
Thin-walled ware	-	-	7 frag	3
Lamp	-	-	1	1
Terra Sigillata		-	-	1
**MNI**	**117**	**70**	**81**	**129**

The discovery of whale remains within the Roman Republican level 19 of Compartment 1 at Castelo Velho de Safara is significant both archaeologically and for the study of historical ecology. This find, situated many kilometres away from the sea, underscores the remarkable mobility and trade networks of this ancient society. The presence of these cetacean remains in an inland location suggests a sophisticated level of organisation, and the capacity for long-distance transportation and preservation of marine resources. Such evidence indicates a trade, or exchange system, that valued such remains either for their material utility or symbolic significance.

## 2. Materials and methods

A systematic zooarchaeological review was carried out to catalogue all cetacean remains from archaeological sites in Portugal, drawing on both published literature and Portuguese zooarchaeological reports. We conducted a thorough search including journal articles, books and conference papers and used academic databases, such as Google Scholar, JSTOR, and specific archaeological journals. We accessed zooarchaeological reports from various Portuguese archaeological institutions, museums and archives. This included reports from past excavations and ongoing research projects. We collaborated with Portuguese zooarchaeologists and researchers who provided access to unpublished reports and shared their expertise. The available information from all chronological contexts is summarised in Table 2 including the site name, location (given that the exact georeferenced coordinates were not always provided in the literature reviewed, we did not include them in the study; instead, we used the general location information available), the type of site (e.g., settlement, pit, garbage dump), the site’s chronology, cetacean taxonomy, skeletal parts present, the Number of Identified Specimens (NISP), the Minimum Number of Individuals (MNI) and the authors’ interpretation of cetacean use (e.g., consumption, use as raw material). The data was organised according to archaeological levels due to the presence of some sites with occupations from various periods. For instance, Castelo de Palmela shows cetacean remains from both a Medieval occupation in the 13^th^-14^th^ centuries and a Modern occupation from the 17^th^ century [24]. It is possible that more sites with cetacean remains exist in Portugal, so this should be considered a non-exhaustive dataset that can always be updated. The results of this review are then put in context based on other archaeological and historical data, in order to ascertain the most probable way of cetacean procurement, whether through active hunting or through scavenging of beached animals.

**Table 2:** Summary of archaeological cetacean finds in Portugal, including site locations, dating information, species and anatomical identifications, contextual and quantification analyses (i.e. the Number of Identified Specimens or NISP; the Minimum Number of Individuals or MNI), the cited authors’ interpretation of cetacean use as well as the used references to gather such information. It serves as a pivotal resource for understanding the breadth and diversity of cetacean presence and human interaction with these marine mammals in Portugal.

Site	Location	Type of Site	Chronology	Species	Anatomy	NISP	MNI	Use	References
Gruta da Figueira Brava	Setúbal	Cave	Middle Palaeolithic	*Delphinus delphis*	6 vertebra	6	1	Consumption	Antunes (2000) [34]
Lagar Velho	Leiria	Rockshelter	Upper Palaeolithic	Cetacean, probably dolphin	Vertebra	1	1	Indeterminate	Moreno-García & Pimenta (2002) [35]
Alpena	Trafaria	Settlement	Chalcolithic	Cetacean	Large flat bone	1	1	Possible board for spreading pigments (tinta)	Cardoso (1995) referring to Zbyszewski (1977) [42, 44]
Castro do Zambujal	Torres Vedras	Settlement	Chalcolithic	Cetacean, probably whale	Bone fragment	1	1	Indeterminate	Schumacher et al (2013) referring to Driesch & Boessneck (1976:96) [39, 43]
Leceia	Oeiras	Settlement	Chalcolithic, pre-Bell Beaker	Cetacean	Proximal rib	1	1	Chopping anvil–with cut marks	Cardoso (1995) [44]
Rotura	Setúbal	Settlement	Chalcolithic, Bell Beaker	Cetacean	Fragment	1	1	Indeterminate	Gonçalves (1971) [41]
Galeria da Cisterna	Torres Novas	Cave	Chalcolithic, Bell Beaker	Sperm whale (*Physeter macrocephalus)*	Bone fragment—maybe teeth	15	Indet	Worked bones—Buttons	Zilhão et al (2022) [40]
Verdelha dos Ruivos	Vila Franca de Xira	Burial—cave	Chalcolithic, Bell Beaker	Sperm whale (*Physeter macrocephalus)*	Bone fragment—maybe teeth	5	Indet	Worked bones—Buttons	Schumacher et al (2013) [39]
Palmela, Quinta do Anjo	Setúbal	Burial—rock cut	Chalcolithic, Bell Beaker	Sperm whale (*Physeter macrocephalus)*	Bone fragment—maybe teeth	2	Indet	Worked bones—Buttons	Schumacher et al (2013) [39]
Dolmen das Conchadas	Sintra	Burial	Chalcolithic, Bell Beaker	Sperm whale (*Physeter macrocephalus)*	Bone fragment—maybe teeth	3	Indet	Worked bones—Buttons	Schumacher et al (2013) [39]
Praia das Maçãs	Sintra	Burial	Chalcolithic, Bell Beaker	Sperm whale (*Physeter macrocephalus)*	Bone fragment—maybe teeth	2	Indet	Worked bones—Cylinder; Bead	Schumacher et al (2013)
Pedra do Ouro	Alenquer	Settlement	Chalcolithic, Bell Beaker	Sperm whale (*Physeter macrocephalus)*	Bone fragment—maybe teeth	5	Indet	Worked bones—Buttons	Schumacher et al (2013) [39]
Monte Molião	Lagos	Settlement	Iron Age	Cetacean	Vertebra	2	1	Indeterminate	Detry & Arruda (2013) [110]
Museu Municipal de Faro	Faro	Settlement	Protohistory/Roman	Cetacean	Vertebra	1	1	With metal bit in the middle of centrum	Veríssimo (2020) [104]
Biblioteca de Mértola	Mértola	Settlement	Roman Republican, 2nd century bC	Cetacean	Vertebra	2	1	Chopping anvil—with cut marks	Moreno-García & Pimenta (2020) [36]
Castelo Velho de Safara	Moura	Settlement	Roman Republican	North Atlantic right whale (*Eubalaena glacialis*)	Bone fragment	1	1	Indeterminate	Current work
Boca do Rio	Vila do Bispo	Villa	Roman Imperial	Cetacean, probably whale	Intervertebral disc	1	1	With hole in the middle of the centrum	Bernal-Casasola et al (2016) [85] referring to personal communication by J.P. Bernardes
Monte Molião	Lagos	Settlement	Roman Imperial	Cetacean	Vertebra	1	1	Worked bones	Detry & Arruda (2013); Detry & Tavares da Silva (2016) [110, 111]
Tróia	Grândola	Fish-salting production	Roman Imperial; workshop 1, UE 488, #7972	Cetacean, probably dolphin	Vertebra	1	1	Indeterminate	Nabais unpublished
Creiro	Setúbal	Fish-salting production	Late Antiquity, 4th and 5th centuries; phase 2—layer 2, tank 2, workshop F14	Cetacean, probably whale	Vertebra	11	1	Chopping anvil—with cut marks	Detry & Tavares da Silva (2016) [111]
Museu Nacional de Machado de Castro	Coimbra	Garbage dump	Medieval	Cetacean	Fragment	1	1	Indeterminate	Silva (2015) [116]
Alcáçova de Santarém	Santarém	Silo/Pit—Layer 1, pit 3	Medieval—Moslem period, 9th to 12th century	Cetacean, probably dolphin	Vertebra (unfused)	1	1	Consumption?	Davis (2006) [108]
Portela 3	Messines	Settlement	Medieval—Moslem period, 10th to 13th century	Whale	Vertebra	1	1	Chopping anvil—with cut marks	Pereira (2015) [115]
Castelo de Paderne	Albufeira	Settlement	Medieval–Moslem period, 12th to 13th century	Dolphin	Vertebra (unfused)	1	1	With cut marks	Pereira (2013) [114]
Silves	Silves	Garbage dump	Medieval—Moslem period, 12th to 13th century	Whale	Vertebra	3	1	Chopping anvil—with cut marks	Davis el al (2008: 206) [109]
Ribat da Arrifana	Aljezur	Settlement	Medieval—Moslem period, 12th century	Cetacean	Bone fragment	1	1	Indeterminate	Antunes (2007: 84; 2011) [106, 107]
Poço Antigo, Cacela-a-Velha	Vila Real de Santo António	Settlement	Medieval—Moslem period, 12th to 13th century	Whale	Vertebra fragments	8	1	Consumption and Chopping anvil—with cut marks	Francisco (2022) [112]
Rua Henrique Calado	Albufeira	Silo/Pit	Medieval—Moslem period, 13th century	*Delphinus delphis*	Vertebra (unfused)	3	1	Cut marks and rodent gnawing	Antunes et al (2012) [102]
Rua Henrique Calado	Albufeira	Silo/Pit	Medieval—Moslem period, 13th century	Cetacean, probably whale	Bone fragment	24	1	11 fragments show blue/white burns	Antunes et al (2012) [102]
Ponta do Castelo	Aljezur	Settlement	Medieval—Moslem period, 12th to 13th century	Whale	Bone fragment	1	1	Used as a bench (close to the fireplace)	Gomes, Assunção, Miranda (2001) [113]
Arronchela	Silves	Settlement	Medieval—Moslem period, 13th to 14th century	*Globicephala melas*	Bone fragment	1	1	Found in structure 8	Gomes (2011: 169) [38]
Castelo de Palmela	Palmela	Settlement	Medieval, 13th-14th century	*cf*. *Delphinus delphis*	Vertebra	2	1	Consumption—with deep cut marks	Fernandes et al (2012) [24]
Avenida Miguel Fernandes	Beja	Silo/Pit	15th century	Cetacean, probably dolphin	Vertebra	1	1	Indeterminate	Detry et al (2021) referring to a personal communication by Moreno-García [37]
Peniche	Peniche	Shipwreck	16th-17th centuries	North Atlantic right whale (*Eubalaena glacialis*)	4 occipital, 10 skull indet, 3 sets of fused cervical vertebra, 11 vertebra indet, 1 verebral disc, 1 ulna, 8 ribs, 42 indet frags	80	3	Consumption	Teixeira et al (2014) [45]
Carnide	Lisboa	Silo/Pit	17th century	*cf*. *Delphinus delphis*	Vertebra (unfused)	1	1	Consumption	Detry et al (2021) [37]
Castelo de Palmela	Palmela	Settlement	17th century	*cf*. *Delphinus delphis*	Vertebra	1	1	Chopping anvil—with cut marks	Detry et al (2021) [37]
Casa das Bicas	Loulé	Garbage dump	18th century	*cf*. *Delphinus delphis*	2 mandibles, 1 vertebra	3	1	Indeterminate	Valente, personal communication

The new evidence recovered from Castelo Velho de Safara (Fig 1) was included in Table 2. These cetacean remains were recovered in 2019, from level 19 in Compartment 1, dated to the early 1^st^ century BCE, based on the associated cultural material recovered from that deposit as described above. The cetacean remains consist of many large fragments of very porous spongy bone that might have been part of a single skeletal part (a possible vertebra), but which started to crumble once removed from the sedimentary deposit. These remains were examined for signs of butchery, burning and other taphonomic marks in the LARC-Archaeosciences Laboratory in Lisbon (Portugal). Species identification of the specimen was not possible using morphological criteria.

Instead, Zooarchaeology by Mass Spectrometry (ZooMS) was applied. ZooMS is an established biomolecular technique that is increasingly being used in the identification of zooarchaeological remains. The method uses the analysis of bone collagen to identify remains to the family, subfamily, genus, or in some cases, species level (depending on the taxa under study; [25]). The amino acid sequences of the collagen peptide that are preserved in the bone can be used to differentiate between related cetacean genera [3, 26–28]. Nonetheless, a minimum evolutionary divergence of 5–6 million years between species is generally required for effective identification using ZooMS [29]. Therefore, ZooMS cannot, for example, differentiate between the bowhead whale (*Balaena mysticetus*) and the North Atlantic right whale (*Eubalaena glacialis*) [26, 30].

A small fragment of roughly 500 mg was removed from the Safara whale bone specimen using a Dremel® rotary tool. Collagen was extracted as detailed in van den Hurk et al (2023b) [10], at the National Laboratory for Age Determination, Norwegian University of Science and Technology, Norway. Roughly 0.1 mg of collagen was taken to the Henry Wellcome Laboratory, University of Cambridge, UK, and treated for ZooMS analysis as detailed in van den Hurk et al. (2023b) [10], and run on a Bruker ultraflex III MALDI tandem flight-of-flight (TOF/TOF) mass spectrometer. The resulting spectra were subsequently analysed using mMass software [31] and visually compared with published *m/z* markers [25, 32, 33]. The ZooMS spectra was made publicly available in the Dryad repository and can be accessed at https://doi.org/10.5061/dryad.zgmsbcch7 Information regarding the ethical, cultural, and scientific considerations specific to inclusivity in global research is included in the S1 File. No permits were required for the described study, which complied with all relevant regulations.

## 3. Results

### 3.1. Temporal and geographic distribution

**Fig 2:**
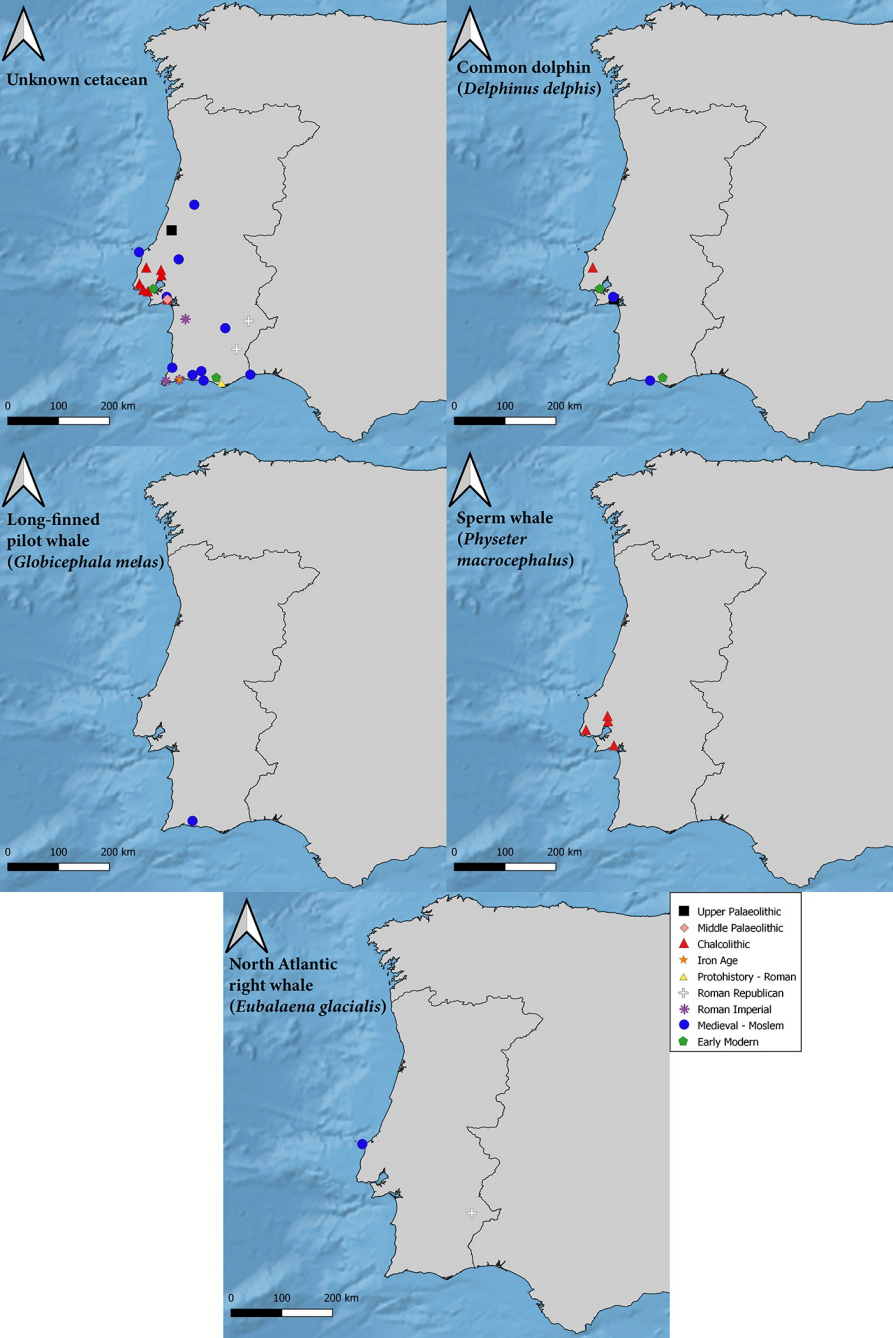
Distribution map of cetacean finds in Portugal. Each point represents a specific site where remains were uncovered, ranging from the Middle Palaeolithic to the 18th century, with distinct symbols indicating the different time periods and cetacean species identified. Please refer to Table 2 for more information on each site.

Cetacean remains have been recorded in numerous archaeological sites all over the Portuguese mainland. The presence of such remains is sparse during the Palaeolithic; they have been found in only two sites: the Middle Palaeolithic cave of Figueira Brava [34], and the Upper Palaeolithic rockshelter of Lagar Velho [35]. So far, no cetacean remains have been reported for Mesolithic or Neolithic levels. However, there is a dramatic increase of cetacean evidence during the Chalcolithic and the Bell Beaker period in particular, followed by a lack of evidence during the Bronze Age (Fig 4). The Chalcolithic remains were mostly found in the Portuguese Estremadura. Cetacean evidence is scarcely reported for Iron Age levels but their frequency increases again with the Roman occupation of Iberia, with most evidence coming from littoral sites, except for Biblioteca de Mértola [36] and Castelo Velho de Safara (Figs 2 and 4).

In later time periods, frequency data shows a clear increase in cetacean remains, mainly during Medieval times ranging between the 9^th^ and the 14^th^ centuries, but most markedly in the 12^th^ and 13^th^ centuries (Table 2). Such cetacean remains are generally associated with Moslem occupations in littoral areas particularly in the Algarve region, in the southern coast of Portugal. The evidence drops after the 14^th^ century, even though there are still remains recovered from sites dated until the 18^th^ century. Once more, the evidence is mainly recovered from coastal occupations, with the exception of the fragment found in Avenida Miguel Fernandes, located inland, in a 15^th^ century pit in Beja [37]. Lastly, it should be noted that all archaeological cetacean remains were recovered from the central and southern areas of Portugal, whereas evidence from the northern parts of the country is yet to be reported (Figs 2 and 4).

### 3.2. Species identification

A total of 196 cetacean remains were found in 37 Portuguese archaeological levels as referenced in published papers and other grey literature (Table 2, Fig 4), including the recent find from Castelo Velho de Safara. More than half of these levels (59.46%, corresponding to 22 archaeological levels) present remains that are not identifiable to species. Such bones are commonly recorded as “dolphin”, “whale” or “cetacean”, sometimes referring to a large or small cetacean, being the large specimens generally associated with whales and small specimens with dolphins. This calls for the need for biomolecular analyses (either ZooMS or aDNA analysis) to optimise species identification on these specimens.

A total of 15 archaeological levels show cetacean remains that were assigned to a taxonomic identification. On six occasions, cetaceans were identified through morphological analysis, mainly those recorded as common dolphin (*Delphinus delphis*, or cf. *Delphinus delphis*). *Globicephala melas* (a species from the Delphinidae family, but known as the long-finned pilot whale) was recovered from structure 8 of Arronchela [38], but there is no indication of the methods used in species identification. Other remains were recorded as “Cetacean, probably dolphin”, or simply as “Dolphin”, leaving it unclear what dolphin species these specimens represent.

A similar extrapolative line of reasoning is not as clear within whales, since two different species have been identified. Whale remains recovered from six sites of Bell Beaker chronology were identified as *Physeter macrocephalus* (sperm whale), using a combination of scientific methods, such as optical microscopy, measurement of specific gravity, measurement of hardness, Micro-Raman spectroscopy, elemental analysis, Isotopic Mass Spectrometry [39, 40]. A more detailed analysis, potentially using ZooMS, is needed to identify the cetacean remains found at the contemporary sites of Rotura, Alpena, Castro do Zambujal and Leceia [41–44].

**Fig 3:**
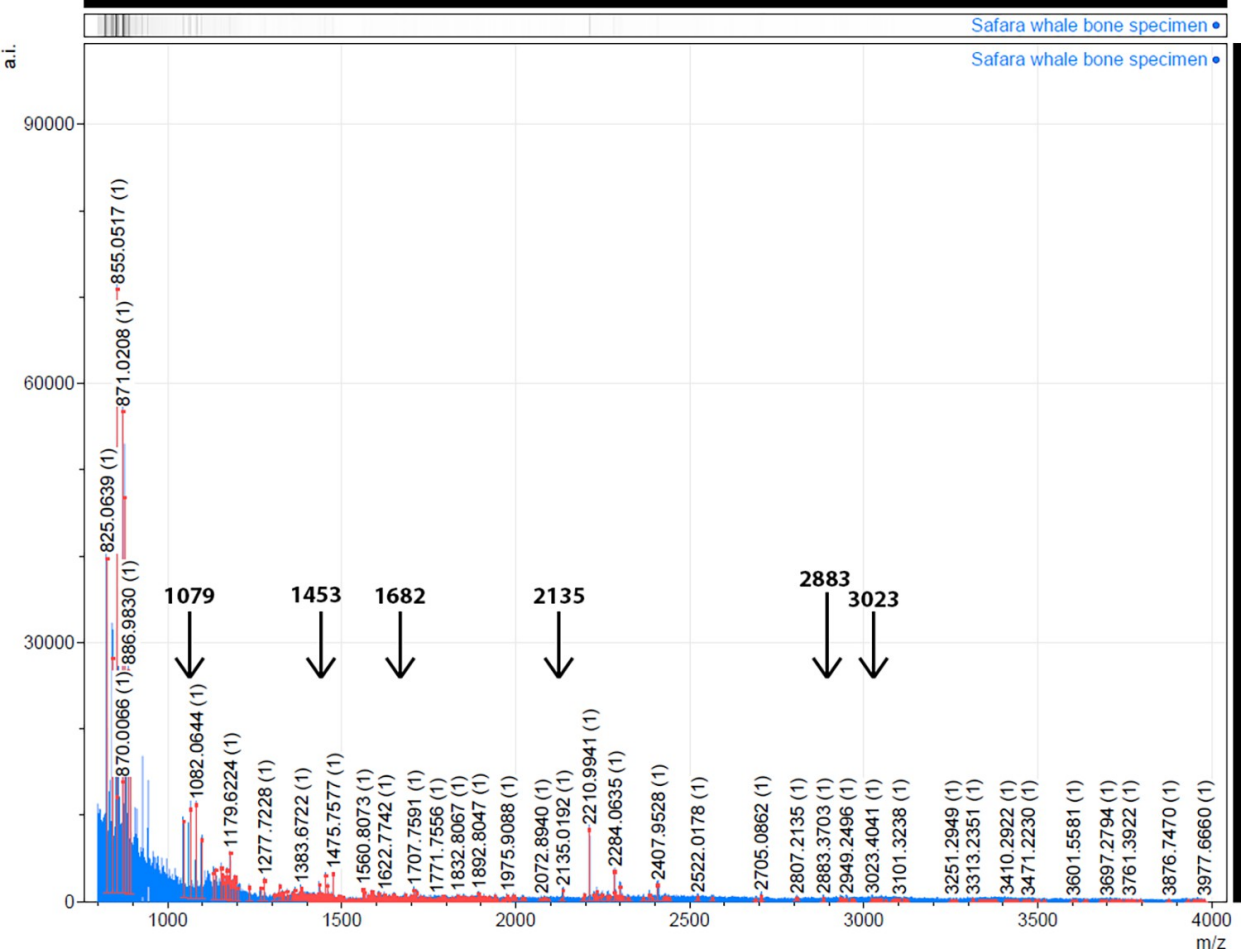
Zooarchaeology by Mass-Spectrometry (ZooMS) analysis for Castelo Velho de Safara’s specimen, highlighting with arrows the diagnostic peaks for Balaenidae.

The large assemblage of North Atlantic right whale (*Eubalaena glacialis*) recovered from Peniche was identified based on morphological skeletal traits. Uncharacteristically, many of the bone remains from Peniche were (relatively) complete, allowing to reach this identification based on the morphology of several cervical vertebral units and one ulna [45]. The same whale species was also determined for the remains found in Castelo Velho de Safara, despite not being able to be separated from the bowhead whale (*Balaena mysticetus*) using ZooMS (Fig 3), and thus being generally classified as Balaenidae. However, and chiefly based on the find location, the North Atlantic right whale is the most likely identification, as the bowhead whale primarily occurs in Arctic waters while the North Atlantic right whale is known to have historically occurred in Portuguese waters [10].

### 3.3. Skeletal part representation and age at death

Most cetacean elements are often not anatomically identifiable. However, among those allowing identification, they are mainly vertebral fragments (NISP = 63, or 32.1% of the total number of cetacean remains listed in Table 2), although there are also mentions to other anatomical elements, such as skull bones (NISP = 14, or 7.1%), ribs (NISP = 9, or 4.6%), mandibles (NISP = 2, or 1.0%), some possible teeth (NISP = 6, or 3.1%), and one ulna. The remaining 74 (or 37.8%) cetacean fragments are recorded as anatomically indeterminate (Table 2).

The vertebrae found in the Medieval occupations of Alcáçova de Santarém, Castelo de Paderne, Rua Henrique Calado, and in the 17^th^ century pit of Carnide have been recorded as unfused. Additionally, there are also two unfused intervertebral discs from Roman Boca do Rio and the 16^th^-17^th^ century assemblage from Peniche (Table 1). These can indicate the presence of young animals, but it should be cautioned that some larger whales can have unfused vertebra up to 30 years old [46]. Conversely, Peniche has also provided three sets of fused cervical vertebrae (the fusion of all seven cervical vertebrae is typical for Balaenidae, such as the North Atlantic right whale), therefore suggesting the presence of at least three individuals. All other sites were only represented by a single individual (Table 2).

### 3.4. Bone surface modifications

Some taphonomic alterations have been reported (Table 2). In the 13^th^ century Rua Henrique Calado, a total of eleven fragments of a probable whale show thermo-alterations related to high temperature exposure of more than 400°C, since the remains present blue and white burns [47].

Cut marks have also been reported for eleven archaeological levels (Table 2). Most of them–i.e. from seven archaeological levels–are interpreted as being related to the use of cetacean remains as chopping anvils. In two cases–in Castelo de Paderne and Rua Henrique Calado–cut marks were found on unfused dolphin vertebrae from Medieval sites in the Algarve, and show no interpretation associated to their possible use. In Castelo de Palmela and in Peniche, cuts seem to be associated with cetacean consumption. Cetacean consumption is also interpreted by researchers for another four levels, even though no marks have been reported (Table 2).

Finally, other marks are related to holes in the centre of vertebral elements, in which a metal bit was probably located, such as the case of Museu Nacional de Faro. One specimen from Monte Molião and most of the Chalcolithic remains are related to bone working activities, being the objects mainly interpreted as buttons. Other explanations of bone modification are related with their use as a board for spreading pigments (in Alpena), or their use as a fireplace bench (in Ponta do Castelo) (Table 2).

## 4. Discussion

### 4.1. Spatiotemporal species distribution

The coast of central and southern Portugal has several important submarine valleys, such as the ones of Nazaré, Lisbon-Setúbal, São Vicente and Portimão [48, 49]. These deep waters close to the shore also benefit from predominant north-easterly trade winds, and from an upwelling system that occurs along the Portuguese mainland coast, resulting in a dynamic ecosystem that has very rich fishing grounds and is highly favourable to cetacean occurrences [50, 51]. These oceanographic characteristics can maybe explain why our review of cetacean remains from Portuguese sites show no archaeological evidence coming from the north of the country. However, this is contrasting with frequent current sightings of bottlenose and common dolphins in northern coastal areas of mainland Portugal, and sightings of some beaked whales that sometimes venture towards northern latitudes [52]. Historical references seem to support this idea of absence of cetacean remains in the north of the country. This is due to the accepted theory of Aguilar (1986 [9]) that active whaling started in the French Basque country in 1059 CE and then spread progressively westwards along the northern coast of Spain, until it got to Galicia in 1371. However, there is no timeline progression of whaling knowledge coming from the Basque country and Galicia to the north of Portugal [53], which led Teixeira et al (2014 [45]) to suggest that whaling in Portugal originated independently of direct Basque influence. Indeed, such whaling activity was most certainly performed on the Portuguese coast before the 14^th^ century since there are at least 38 historical accounts of scavenging of stranded animals and whaling between 1201 and 1728 CE [45]. So, if that is the case, then whaling in Portugal started in the central and southern parts of the country.

**Fig 4:**
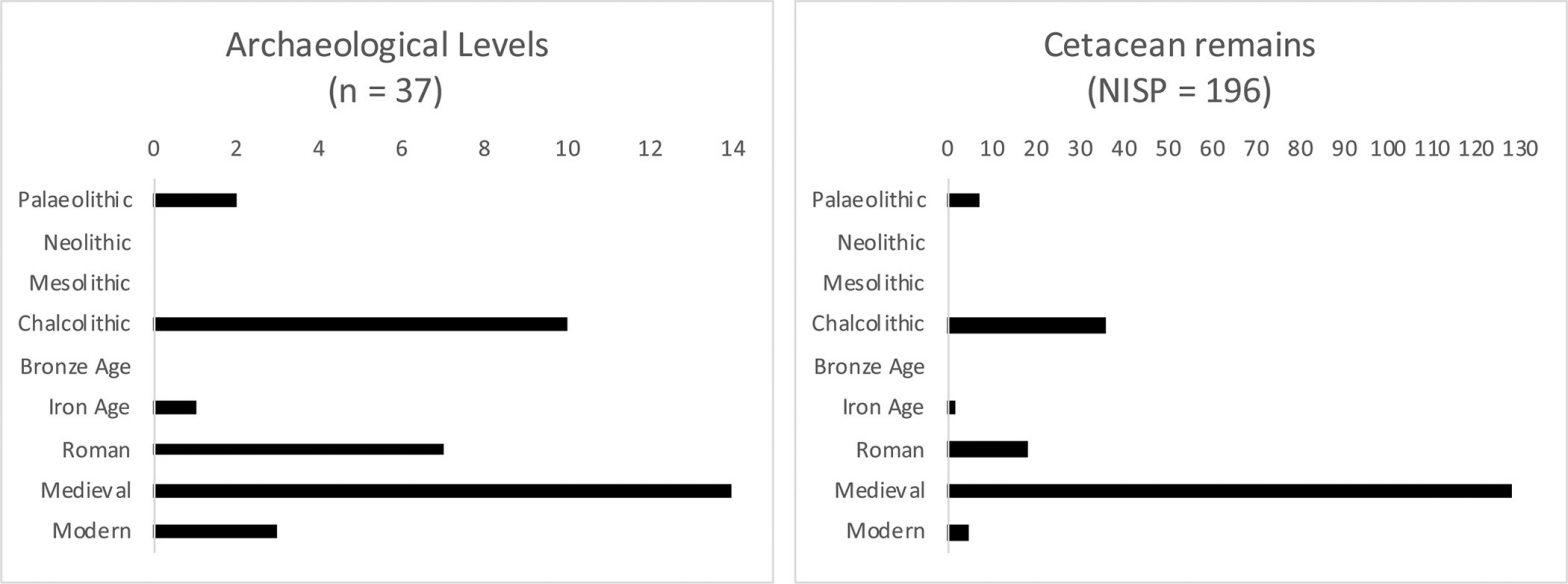
Comparison of cetacean remains across different Portuguese archaeological periods, showing the number of archaeological levels that display cetacean remains alongside the Number of Identified Specimens (NISP) for each period. This figure highlights the distribution and quantitative analysis of cetacean remains illustrating temporal trends.

Scavenging of stranded cetaceans was carried out since the Palaeolithic, as shown by Gruta da Figueira Brava [34] and Abrigo do Lagar Velho [35]. This early human behaviour is generally thought to have been opportunistic, taking advantage of natural events where marine mammals became stranded on shore, easily accessible to human populations of that time. It is unexpected, however, to find no reports on cetaceans from the somewhat vast evidence of Mesolithic sites, when Portuguese human populations had a very close relationship with the shore and aquatic resources [54]. The absence of evidence continues into the Portuguese Neolithic. However, during this period, active exploitation of marine mammals was already occurring in the, so far only known example of contemporary sites, coastal Vlaardingen Culture, in current western Netherlands [10, 55]. The scenery is highly contrasting with the Chalcolithic evidence, which presents ten archaeological levels showing a total of 36 cetacean remains (Table 2, Fig 4). Most of them were carved into buttons made of sperm whale bones. This species is abundant in the Mediterranean, and in the strait of Gibraltar in particular [56]. They are very large animals and their pursuit was considered to be dangerous, which is reflected on their late active exploitation that may have not started until 1713 CE [57]. Therefore, sperm whale bones had an important significance to Chalcolithic people, and these carved sperm whale bones may be evidence for an integrated trading route system. They may have been originally obtained from Mediterranean shores, and then reaching central Portugal, sometimes arriving to inland positions, such as the case of Galeria da Cisterna [40]. These trades may have existed since the Upper Palaeolithic considering that whale bones from eleven sites dating to the era were found in the Spanish Pyrenees, implying transport of cetacean remains from the Mediterranean and/or the Atlantic coasts of Iberia [58, 59]. This is also the case of the cetacean remain found in Lagar Velho [35]. Such trading routes may have endured over time, since whale remains are found in inland Portugal during the Roman Republican occupations of Castelo Velho de Safara and of Biblioteca de Mértola [36], as well as in the 15^th^ century levels of Avenida Miguel Fernandes [37]. If cetacean bones were considered to be a valued product, they may have been traded to distant regions and preserved for many years [60].

Other than accommodating sperm whales, the central and southern Portuguese shores may have lodged North Atlantic right whales, with confirmed archaeological representation in Castelo Velho de Safara and Peniche [45]. Genetic evidence [61] and historical documentation [53, 62] and references therein] indicate that the whales hunted along the shores of Portugal were likely North Atlantic right whales. Calving and/or migrating right whales would have been reliably found close to the coast before they were overexploited leading to only few individuals surviving into the late 17^th^ century and the species becoming extirpated from eastern North Atlantic waters probably somewhere in the mid-20^th^ century [9, 10]. The Portuguese central and southern coasts are in similar latitudes to those where these whales calve nowadays in the western side of the North Atlantic, and right whales are one of the most frequently found species in the European archaeological record [10, 28, 63–65].

### 4.2. Whaling

Despite the relatively small proportion of cetacean bones generally found in archaeological sites, their type of procurement has been debated for some time [e.g. 57, 66]. Human exploitation of marine mammals in Portugal started in the Middle Palaeolithic, with evidences coming from Gruta da Figueira Brava [34]. They refer to remains of common dolphins, which have been resident in the river Sado estuary to this date. Even today, common dolphins get stranded due to marine currents or sudden tide movements. In such circumstances, these individuals become an easy target for human predation. This strategy was also the most probable for the Chalcolithic period. As described above, Chalcolithic cetacean remains are mainly represented by sperm whales, whose capture only started in the Modern era [57]. Additionally, there have not yet been identified any objects from the Chalcolithic, or the early Bronze Age, in Iberia that are interpreted as harpoons, or that could have been used as such [39]. This echoes Dutch evidence on whale remains, with very few findings coming from the Bronze Age period [67].

Given the rarity of cetacean remains in archaeological sites, it has been proposed that their use and exploitation were merely opportunistic [68]. However, the scarcity of bones is not incompatible with active cetacean exploitation, especially during periods of known use of boats, fishing gear, and marine resource consumption. In Iberia, it is suggested that active whaling started in the late Bronze and early Iron Ages [69, 70]. This becomes clearer in sites dating from the Roman period onwards. Although not discarding opportunistic exploitation of stranded animals, the technological sophistication in terms of fisheries and seafaring is undeniably compatible with active whaling [71]. This is supported by classic literature, such as Pliny’s description of an attack of an orca that entered the Port of Ostia, “Cæsar ordered a great number of nets to be extended at the mouth of the harbour, from shore to shore […] boats assailed the monster, while the soldiers on board showered lances upon it” (*Naturalis Historia* 9.14; [72]); or the Oppian’s description of the capture of a sea-monster, “(…) the fishers allow him all the length of the line; for there is not in men strength enough to pull him up and to overcome the heavy monster against his will” (*Haliaeutica* V; [64, 73]). Due to their location and functionality, it is possible that the Roman fish-salting industries found in Tróia and Creiro could perhaps have included cetaceans in their fishing activities, akin to what is suggested for Baelo Claudia in the south of Spain [56]. Cetaceans were historically hunted in the Sado estuary, encompassing Setúbal and Sesimbra, during Medieval and Modern times, with industrial whaling operations occurring in the Setúbal area in the early 20^th^ century [74, 75]. Between 1926 and 1927, the river Sado estuary was exploited by the “Portuguese Cetacean Society”. In those two years, a total of 372 fin whales, 55 sperm whales and one indeterminate whale were captured. These animals were processed in the large facilities built in Tróia, which included a 320-metre berth [76].

Literary sources become more helpful regarding Medieval periods. According to Brito and Sousa (2011 [77]), a total of sixteen cetacean laws were passed between the 12^th^ and 21^st^ century, in Portugal. Some Medieval examples show how laws regulated the capture and use of cetaceans, as well as the distribution of their spoils, therefore confirming that an active whaling exploitation was fruitful and already well established. Some examples are those of the charters (or “forais”, in Portuguese) conceded to cities in the south of Portugal by king D. Afonso III (in 1266) and king D. Manuel I (in 1504), both assuring the king’s property of all whales and other “royal fish” [78]. Other examples come from the description of taxes related with trading of whale products. In 1206, king D. Afonso II fixed high tax values for all cetacean goods from Atouguia’s seaport (in central Portugal, near Peniche). Again, in 1510, a new charter was conceded by king D. Manuel I to the same area, listing all economic activities that should be taxed, which included marine trade and fishing [45]. Whaling was highly profitable for the Portuguese kingdom, aligning with an increase in archaeological evidence from the Medieval period. This period accounts for 37.8% of the archaeological levels containing cetaceans, and 65.3% of all cetacean remains found, largely influenced by the significant finds from the Peniche shipwreck (Table 2, Fig 4).

The presence of possible juvenile cetaceans found in the Portuguese archaeological samples could maybe indicate a propensity to strand in younger and inexperienced animals due to their ease of handling. Moreover, a mother-calf pair could have been targeted, as the mother would not leave her calf behind, leading to the mother becoming an easy target as well. Cetaceans’ sightings from the coast in Medieval and Modern times were regular, since such marine populations were ten times greater than today and, hence, more cetaceans would inevitably be available for human hunting [77, 79]. As noted by Teixeira et al (2014 [45]), Portuguese references to whaling techniques are fairly inconclusive, but they surely used simple technology, which included lookout spots in high places on land, small open boats and hand-thrown harpoons [80]. Nonetheless, these basic techniques allowed overexploitation of these large marine mammals, likely contributing to one of the two whale historical extirpations in Portugal, the North Atlantic right whale [the other being the grey whale, *Eschrichtius robustus*, though findings of this species have not been reported in Portugal; evidence from all over Europe suggests the species was once present in Portuguese waters [10]]. This likely happened somewhere in the last couple of centuries [9, 45].

The final European catches of the North Atlantic right whale occurred in Portugal. In February 1967 a mother-calf pair was taken in Madeira, with a male individual which was accompanying them, being able to flee [81]. A pregnant female was also taken in Madeira in January 1959 [82]. The only sighting in mainland Portugal of this species since then was in 1995, even though it most probably referred to two wandering individuals from the western North Atlantic population, and not remnants of the eastern North Atlantic population [83]. Nonetheless, cetaceans continued to be hunted during the 20^th^ century, shifting the target towards the fin whale, which was mainly captured off the continental shelf of the southwestern coast [45]. It was only in 1981 that the first law of marine mammal protection and preservation was passed in Portugal [77].

### 4.3. Cetacean use

Assessing the function of archaeological cetacean remains is problematic, since they are often found in isolation and are normally damaged. Additionally, cetacean carcasses can have a number of uses, since they provide skin, meat, blubber/oil and bone, which can be used for consumption, illumination and in a myriad of artefacts, architecture and as fuel [45, 84].

Chopping boards, primarily made from cetacean vertebrae, appear to be one of the most common cetacean-related artefacts found in the archaeological record [85]. Similar examples have been discovered in The Netherlands, England, Scotland, and various other regions [10, 30, 65, 86–88]. Portuguese archaeological sites with cetacean remains follow a similar trend. Given the lack of chronological confirmation of the red pigment found on the Alpena vertebra [44], it is possible that this artefact could have been a blank for a chopping board. In the few instances when a taxonomic identification is attempted for chopping anvils recovered from Portuguese sites, whales are the animals typically mentioned. These chopping blocks were mainly made from large vertebra or large flat bones (e.g. Leceia or Alpena), likely associated with large cetaceans. Dolphins, providing smaller bones, are generally unsuitable as raw materials [84]. When consumption is proposed at Portuguese sites, it is often related to dolphins, except for the whales found in Peniche. Based on this evidence–chopping blocks are normally associated with whales, and dolphins are more frequently used for food–we propose that the dolphin remains identified on the 17^th^ century levels of Castelo de Palmela are most likely related to consumption, similar to the interpretation of the 13^th^-14^th^ century levels of the same site.

The high presence of chopping anvils does not discard the possibility that these bones were initially the remains of meals. Although there is no evidence of skeletal part removal for joints of meat to be brought to site, Savelle and Friesen’s (1996 [89]) work on harbour porpoises showed that vertebrae ranked third on a meat utility index. Given this, and the presence of cetacean vertebrae at inland sites such as Biblioteca de Mértola and Avenida Miguel Fernandes, it is plausible that similar anatomical parts were found in the inland settlement of Castelo Velho de Safara. Vertebrae are relatively porous, fitting the characteristics of the cetacean remains found in Safara, although we cannot yet confirm this anatomical identification. If these vertebrae were transportatedinland as meat joints rather than just bones to be transformed into artefacts or tools, the meat would need to be preserved, possibly using salt. Bernal-Casasola (2010 [56]) proposed that some facilities of Roman fish-salting production centre of Baelo Claudia (Cádiz, southern Spain) could have been used to salt whale meat. It is further suggested that such a product could have been marketed in amphorae, supported by inscriptions on Italian amphora type Dressel 21/22 referring to *ceti*, known to be a large species of fish. Coincidentally, or not, these amphorae types had one of their production centres in Algeciras (not far from Baelo Claudia), during the late Roman Republican period and the early empire [56, 90], which match the chronology of the whale remains found in Castelo Velho de Safara.

Akin to the idea of Iberian Roman markets selling whale meat–which appears to have been accessible to people of different social strata–, some Medieval sources indicate a comparable trend in London markets [91]. Although cetacean meat was perceived as a high-status food during Medieval times, usually associated with clergymen and nobility, it was not restricted to these social groups. People of lower social status also had access to it, hoping to mimic the lifestyle and diet of nobility [91, 92]. With the expansion of Christianity and cetaceans being considered animals of the sea, they were classified as fish during the Medieval period [despite Aristotle recognising them as mammals as early as 350 BCE]. Consequently, cetacean meat was deemed acceptable fasting food and was frequently consumed during periods such as Lent [93]. From the early 17^th^ century, Portuguese references show that the prestige of whale meat decreased, and it began to be perceived as ordinary food, consumed primarily by poor people and slaves [94, 95] and historical references therein].

Oil can also be extracted from cetacean blubber and bone. Bernal-Casasola and Monclova (2011 [96]) suggest that the presence of spongy and light inner parts of cetacean bones–similar to those found in Castelo Velho de Safara–could indicate such a practice. Bones could have been boiled to extract fats and oils, but such proposal still lacks archaeometric confirmation [97]. However, it is known that cetacean oil was used for illumination in oil lamps during the Medieval period [98]. Furthermore, whale oil was the main driver for whaling in Portugal and Brazil since at least the 15^th^ century [99]. By the mid-18^th^ century, whale oil was the key fuel used to illuminate the most important urban centres in Portuguese America, and thousands of barrels of whale oil were exported from Brazil to Lisbon for this purpose [95]. Until the discovery of petroleum in 1859, whale oil was of utmost importance. As Eric Jay Dolin (2007 [100]) stated, it “lit the world”.

Cetacean bones could also have been used as fuel. Hambrecht and Gibbons [101] proposed this for a site in Iceland. This is likely the most probable interpretation for the burnt sample recovered from the Medieval levels of Rua Henrique Calado [102], due to their blue and white colouration, which indicates exposure to temperatures higher than those generally associated with cooking activities [47].

Cetacean bone is strong and resilient, making it suitable for artefact production [84]. It could have served as a substitute for the preferred elephant ivory used by Chalcolithic people, with the advantage of a higher bone hardness compared to elephant ivory [39]. Although sperm whale teeth are suggested as the material for carving Portuguese Chalcolithic buttons, other skeletal parts, such as mandibles, might also have been used, since they are composed entirely of solid cortical bone [103]. According to Mulville (2002 [84]), mandible bone is the preferred skeletal part used by the Maori carvers for bone working. Finally, pierced vertebrae or vertebral discs are often recovered from western European archaeological sites, similar to what was found in the Portuguese Roman sites of Boca do Rio [85] and Museu Municipal de Faro [104]. These have been interpreted as “pot lids”, possibly by comparison with those generally made of slab [105 cited by 84].

## 5. Conclusions

Though sparse, cetaceans are present in Portugal since the Middle Palaeolithic, even if sometimes invisible in some periods, such as the Mesolithic, Neolithic and Bronze Age. In Prehistoric periods, cetaceans were most probably exploited opportunistically through scavenging of beached or stranded animals. In historical periods, although such stranded animals were not ignored, active organised whaling practices were in place, as largely supported by several written sources. Cetaceans have been targeted for food, but also for other products, like oil, fat, skin and bone. The latter was particularly important for carving tools and artefacts, and oil became indispensable to illuminate houses and urban centres. Cetacean products have been traded, reaching inland sites since the Palaeolithic until the 20^th^ century. This is particularly evident in Castelo Velho de Safara, where the application of Zooarchaeology by Mass-Spectrometry (ZooMS) for species identification revealed the presence of the North Atlantic right whale within Roman Republican levels. The recovery of whale remains many kilometres away from the sea, not only provides insight into the exploitation and use of cetaceans in these ancient times, but also contributes to a broader understanding of historical patterns of human mobility, trade, and cultural exchange extending far beyond coastal areas.

Cetaceans were so intensively exploited that the North Atlantic right whale, the most common whale species in the Portuguese waters, eventually was extirpated from Portuguese waters. Such misfortune, however, did not prevent the continuation of heavy predation on marine mammals until 1981, when the first protection and conservation law was passed by the Portuguese government. Today, several cetacean species are still resident or regular visitors to the Portuguese waters, but instead of being preyed upon, cetaceans are being increasingly protected, and whale watching has been providing a great economic incentive to preserve their natural habitats.

## Supporting information

S1 File Inclusivity in global research questionnaire file. (DOCX)
